# Indoor and ambient air pollution dataset using a multi-instrument approach and total event monitoring

**DOI:** 10.1038/s41597-025-05847-3

**Published:** 2025-09-29

**Authors:** Mario Lovrić, Valentino Petrić, Dejan Strbad, Teo Terzić, Sanja Frka, Ana Cvitešić Kušan, Jose Fermoso, Sebastian Düsing, Honey Dawn Alas, Mila Dobrić Ladavac, Ivan Bilić, Marko Batrac, Simonas Kecorius, Gordana Pehnec, Tajana Horvat, Ivana Jakovljević, Nikolina Račić, Ivan Bešlić, Darijo Brzoja, Vesna Gugec, Maria Figols, Xabier Aláez, Hana Matanović, Anđelko Žigman, Michael Forsmann, Anneli Toomis, Jürgo-Sören Preden, Alessandro Battaglia, Ivano Battaglia, Gianna Karanasiou, Frederik Weis, Jon Switters, Francesco Mureddu

**Affiliations:** 1 Lisbon Council, 1040 Brussels, Belgium; 2 Institute for Anthropological Research, 10000 Zagreb, Croatia; 3 Ascalia d.o.o., 40000 Čakovec, Croatia; 4 Ruđer Bošković Institute, 10000 Zagreb, Croatia; 5 CARTIF Technology Center, 47151 Boecillo, Valldolid Spain; 6 Leibniz Institute for Tropospheric Research, 04318 Leipzig, Germany; 7 Kobis d.o.o, 10000 Zagreb, Croatia; 8 Institute of Epidemiology, Helmholtz Zentrum München, 85764 Neuherberg, Germany; 9 Environmental Science Center, University of Augsburg, 86135 Augsburg, Germany; 10 Institute for Medical Research and Occupational Health, 10000 Zagreb, Croatia; 11 Croatian Meteorological and Hydrological Service, 10000 Zagreb, Croatia; 12 inBiot Monitoring SL, 31192 Mutilva, Navarra Spain; 13 SMART SENSE d.o.o., 10000 Zagreb, Croatia; 14 COPSAC, Copenhagen Prospective Studies on Asthma in Childhood, Herlev and Gentofte Hospital, University of Copenhagen, 2820 Gentofte, Denmark; 15 Thinnect, 11624 Tallinn, Estonia; 16 Labservice Analytica srl, 40011 Anzola Dell’Emilia, Italy; 17 WINGS ICT Solutions, 17121 Athens, Greece; 18 Palas GmbH, 76187 Karlsruhe, Germany

**Keywords:** Atmospheric chemistry, Scientific data

## Abstract

Indoor air quality (IAQ) significantly influences human health, as individuals spend up to 90% of their time indoors, where air pollutants can accumulate and interact dynamically. Despite advancements in monitoring technology, challenges remain in capturing the temporal and spatial variability of pollutants and understanding the interaction between indoor and outdoor environments. This study addresses these gaps by introducing a comprehensive dataset from a controlled experimental room in Croatia, leveraging a multi-instrumental approach to monitor IAQ across various real-life scenarios. The dataset integrates measurements from low-cost sensors, reference-grade devices, and auxiliary systems to track pollutants such as particulate matter (PM), black carbon (BC), volatile organic compounds (VOC), and indoor events deemed relevant for the assessment of pollutant levels. Key experiments simulated household activities, including cooking, cleaning, human presence, and ventilation, capturing their impacts on IAQ with high temporal resolution. The resulting dataset comprises over 19 subsets. This work contributes to the Horizon EDIAQI project, supporting the development of evidence-driven strategies to improve IAQ.

## Background

People spend considerable time indoors, especially given the shifts in their line of work. Previous research showed that in developed countries, people spend up to 90% of their time indoors^1,2^. Lee *et al*.^3^ showed that low indoor air quality (IAQ) was associated with 1.8 million deaths and more than 60 million disability-adjusted life years globally. IAQ is influenced by numerous factors and sources, including levels of ambient (outdoor) pollution, transport of pollutants between the two media using (natural) ventilation, internal emissions, chemical reactions between gases and particles or surfaces, and dynamic processes like resuspension, deposition, evaporation, growth, and coagulation^4–8^. Ventilation is assumed to be an important contributor to IAQ^9^. Measuring natural ventilation is a rather difficult task, as trace gases and empty houses are needed to run the studies. Matthews *et al*.^10^ showed that cooking generates aerosols that spread throughout the house, often accumulating more on upper floors. Besides ventilation, a significant contributor is indoor air chemistry^4,11,12^. Among the most prominent pollutants are volatile organic compounds (VOC) and polycyclic aromatic hydrocarbons (PAH), which are the most notable^13^. Although substantial research has been conducted on IAQ, there are still limitations regarding temporal and spatial scales when examining pollution sources, indoor-outdoor interactions, and ventilation/filtration. Few studies have explicitly focused on ultrafine particles (UFP), black carbon (BC), submicron particles, and chemical pollutants^14–16^. Variations in ventilation habits, surface materials, and meteorological conditions limit the practical application of stationary, static IAQ investigations^6,16^. Given the difficulty of assessing air quality, low-cost sensors are becoming increasingly popular^17,18^ but are limited in their assessment and deemed as indicative measurements^19^. Detailed sensor validation is often infeasible, ignored, or undeclared. A study by Manu and Rysanek shows^20^ a co-location experiment at scale, with 87 low-cost IAQ monitors, to verify their performance regarding total volatile organic compounds (tVOCs), particulate matter 2.5 (PM_2.5_), carbon dioxide (CO_2_), temperature, and relative humidity. Their results showed that CO_2_, temperature, and humidity sensors reliably met manufacturer specifications, while tVOC sensors had significant accuracy issues, while PM_2.5_ sensors were more consistent compared to other pollutants. Within the Horizon EDIAQI project (Evidence Driven Indoor Air Quality Improvement)^13^, we aim to create a large-scale data collection system that allows science to apply novel algorithms and models to indoor air data to investigate sources and improve our understanding of the issues. In this work, we examine a broad spectrum of pollutants using numerous instruments from several providers to understand household events and instrument quality by leveraging machine learning (ML) or artificial intelligence (AI). Such a data set can be used to classify pollution events and understand contributors to IAQ.

## Methods

This section provides a detailed description of the room where the experiments were conducted and the experiments themselves. It also describes all instruments used for measurements and their positions within the room.

### Room setup

**Fig. 1:**
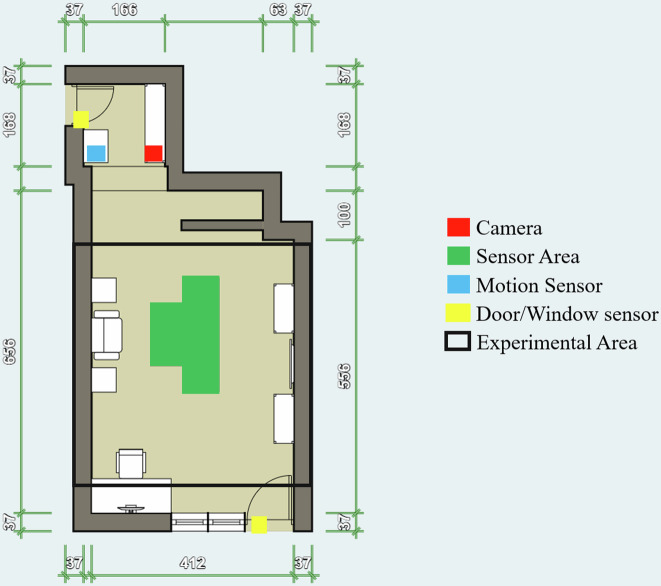
Floor plan of the experimental room.

The measurement campaign was conducted in a single room over 20 days, specifically from October 11, 2024, at 13:20 to October 31, 2024, at 11:45. The room is located on the ground floor of a family home in Sesvetski Kraljevec, east of Zagreb, Croatia. The house is located 4 km from highway E65 and is surrounded by residential buildings. Additionally, no primary industry is present within a 1-square-kilometer area. The distance to a state road is appr. 200 meters. A large forest lies 200 meters opposite the road. Figure 1 shows the room in the house and provides the exact measurements of the room, with all dimensions specified in centimeters (cm). The room has two entrances: one in-house, leading to the hallway, a staircase to the first floor, and the other to the garden. Next to the garden door, there are two sealed windows.

The room was left furnished as initially decorated by the homeowners, with several bookshelves, storage shelves, and a desk with a workstation, as this room is occasionally used as an office. The experimental room area was 29.09 square meters and had a volume of 85.81 cubic meters. The exact measurements of the room are shown in Fig. 1. The figure displays the room’s floor plan with measurements of the walls, windows, and doors. In addition to the measurements, the areas where the sensors are placed are also shown. All sensors used for this study were placed on the table in the middle of the room (indicated by the green area in Fig. 1). The height of the table was 70 cm. The yellow area on the floor plan shows the positions of the door sensors. As the windows could not be opened, no sensors were installed there. Given that the windows and doors are high-end joinery, we assume an insignificant outdoor ventilation rate when the door is closed. The blue area indicates the motion sensor locations, which were placed there to detect the presence of humans. The camera was positioned in the red area of the floor plan, facing the experimental area. All air quality sensors were placed in this experimental area, except for the car experiments conducted outside this location at 3 and 5 meters in front of the garden door.

### Low-cost sensors and instruments

This study used low-cost sensors (LCS) from multiple providers (PRVD) with several experimental instruments under development. Seven different LCS types were employed for indoor spaces, with two models specifically designed for outdoor use. These sensors utilize various technologies to determine indoor air quality conditions and measure air pollutants, with each sensor model employing a unique set of methods. The overview of different data acquired is listed in Table 1, focusing mainly on LCS but also providing auxiliary information for this study, described in the coming sections. A full table, however, is available in the data repository linked to in Section 2.4, which shows each parameter measured in this study, its unit of measurement, and the number of devices each sensor manufacturer provides. The name of each file in which LCS data is stored is also offered. The providers are noted as PRVD1–6 (one to six).

**Table 1:** A table of all files, analytes, and parameters in this study.

File	Description
People.csv	Records of people present inside the room with timestamps
Motion.csv	Motion sensor activations with timestamps
Door_windows.csv	Door opening and closing events with timestamps
Traffic_data.csv	Traffic observations with timestamps
Satellite_data.csv	Satellite data downloaded from the Climate Data Store
Air_flow_data.csv	Speed of air inside the room – small experiments
Chromatography_VOC_measurement.xlsx	Results of chromatographic analysis
7_day_particle_count.xlsx	Seven-day particle analysis from PMs
Black_carbon_data.csv	Black carbon data with timestamps
CPC_data.csv	CPC data with timestamps
Indoor_sensor_provider_1.csv (PRVD1)	Indoor LCS data with timestamp
Indoor_sensor_provider_2.csv (PRVD2)	Indoor LCS data with timestamp
Indoor_sensor_provider_3.csv (PRVD3)	Indoor LCS data with timestamp
Indoor_sensor_provider_4.csv (PRVD4)	Indoor LCS data with timestamp
Indoor_sensor_provider_5.csv (PRVD5)	Indoor LCS data with timestamp
Indoor_sensor_provider_6.csv (PRVD6)	Indoor LCS data with timestamp
Oudoor_sensor_provider_1.csv	Outdoor LCS data with timestamp
Oudoor_sensor_provider_2.csv	Outdoor LCS data with timestamp
Oudoor_sensor_provider_2_ambient_noise.csv	Outdoor noise with timestamp
Outdoor_Zagreb_data.csv	Data from a measuring station close to the measurement site
Indoor_referent_device.csv	Indoor referent device with timestmap

For carbon dioxide (CO_2_) measurement, five out of six LCS providers use the Non-Dispersive Infrared (NDIR) technology^21^, while the sixth provider (PRVD6) employs the Multi-Pixel Metal Oxide (MOx) technology^22^. NDIR technology uses infrared lights to excite chemical compounds inside an air sample tube. A detector at the end of the tube measures the amount of infrared light passing through, which correlates with the CO_2_ concentration in the air. In contrast, MOx sensors allow compounds to react with the metal oxide in the sensor array, causing a change in resistance, from which the gas concentration is determined. For TVOC (Total Volatile Organic Compounds) measurement, all six sensor providers use MOx sensors. For PM measurement, the sensors in these LCS utilize laser particle sensors based on light scattering technology^18^. This method involves lasers interacting with airborne particles, causing light scattering based on the particles’ size and shape. All LCS in this study use this technology for PM measurement. For ozone (O_3_) measurement, an electrochemical reaction occurs within the sensor, where ozone is reduced to oxygen and oxygen ions. This reaction generates an electric current proportional to the ozone concentration in the air. PRVD4 and PRVD5 implement this technology. Additionally, one sensor from PRVD2 uses similar electrochemical technology to detect formaldehyde. The current generated by this reaction depends on the formaldehyde concentration in the air. Electrochemical technology, like ozone and formaldehyde, for CO measurement, detect carbon monoxide (CO) in the air. PRVD4 and PRVD5 implement this technology. PRVD4 can also detect nitrogen dioxide (NO_2_), achieved using electrochemical technology. Three sensor providers use general digital sensors for ambient conditions, while PRVD2 specifies using a Silicon Bandgap Temperature Sensor. This type of sensor measures temperature by detecting resistance changes caused by temperature variations. For humidity measurements, the sensor relies on capacitive changes; as humidity increases, the capacitance also increases. Provider 4 employs a BME sensor for all ambient measurements. Lastly, outdoor devices use the same technology as their indoor counterparts, as the same manufacturer produces them.

### Data acquisition

Data acquisition from the LCS was done using the platform provided by each sensor manufacturer (proprietary software). The LCS was connected to the internet via Wi-Fi or mobile internet connections (SIM cards), and all the data is stored on the platform, which can then be downloaded using the Application Programming Interface (API) or through the platform interface. The data from the LCS are stored in the provided CSV files on personal computers. Depending on the LCS provider, devices had different measurement cycles, ranging from 5 seconds to 5 minutes. Data from motion and door sensors were collected using a Home Assistant (Home Assistant. Retrieved February 3, 2025, from https://www.home-assistant.io) and a Raspberry Pi (Version 4, Raspberry Pi Ltd, Cambridge, UK). The Home Assistant integration allows devices to connect and collect data via Bluetooth. The other data was stored in CSV format, with timestamps marking when the sensor state changed. The camera for human motion inside the room captured a frame every 1 minute, and simple object detection was run using a pre-trained YOLOv8 model^23^. The number of people inside the room was stored alongside the timestamp. This system was run on a Raspberry Pi, and the results of the detections were stored there. In addition to monitoring human motion inside the room, vehicles passing by the house were tracked.

A Reolink (https://reolink.com/) camera captured car motion, and any detected video was stored on the Reolink Cloud platform. The videos were then downloaded and analyzed using a pre-trained YOLOv11 medium model, which detected four classes: car, truck, bus, and motorcycle.

A state-of-the-art referent AQ instrument, the Palas AQ Guard (Palas GmbH, Karlsruhe, Germany), was calibrated before the study and used to compare the data from the low-cost sensors. The data from the Palas device was stored in its internal memory, taken manually, and processed into a CSV file. Alongside generic pollutants (e.g., PM_10_), The Palas AQ Guard has also reported particle number size distribution, a measure valuable to determine smaller fractions of PM. For BC, we used an aethalometer (model AE33, Magee Scientific, USA), which was installed to measure aerosol light-absorption coefficients (b_abs_) at seven wavelengths (370–950 nm) with a time resolution of 1 min. Sampling was performed at a constant flow rate of 5 L min^−1^ using the PM_2.5_ size-selective inlet. The gradual collection of absorbing aerosols on the Teflon-coated glass fiber filter (M8060 filter tape) increases light attenuation. The equivalent BC concentrations were determined from the change in optical time-resolved light attenuation at 880 nm using the mass absorption cross-section (σ_air_) of 7.77 m^2^ g^−1^ and the multiple scattering parameter (C) of 1.39^24^. The dual-spot technology provides an online correction for the filter loading effect^25^. To estimate the contribution of biomass burning (BC_bb_) and fossil fuel combustion (BC_ff_) to BC concentration, a source apportionment method (Aethalometer model) was applied^26^. The model is based on the wavelength dependence of b_abs_, defined by the absorption Ångstrom exponent (α), assuming that the entire aerosol light absorption is the sum of the fossil fuel and biomass combustion fractions. Calculating the α value involves a power-law wavelength dependence of b_abs_, as described elsewhere^27^. A Condensation Particle, CPC (Model 3750, S/N: 3750212702), manufactured by TSI (TSI Incorporated, Shoreview, MN, USA), was used during a two-week measurement period. The CPC operates with a d50 cutoff size of 10 nm and a maximum detectable particle size greater than 3 µm. It features a concentration range of up to 100,000 particles/cm³ in single particle counting mode, with an accuracy of ±5% for concentrations below 100,000 particles/cm³. The device is equipped with an internal water removal pump, making it suitable for high-humidity environments, and it uses butanol as the working fluid.

**Fig. 2:**
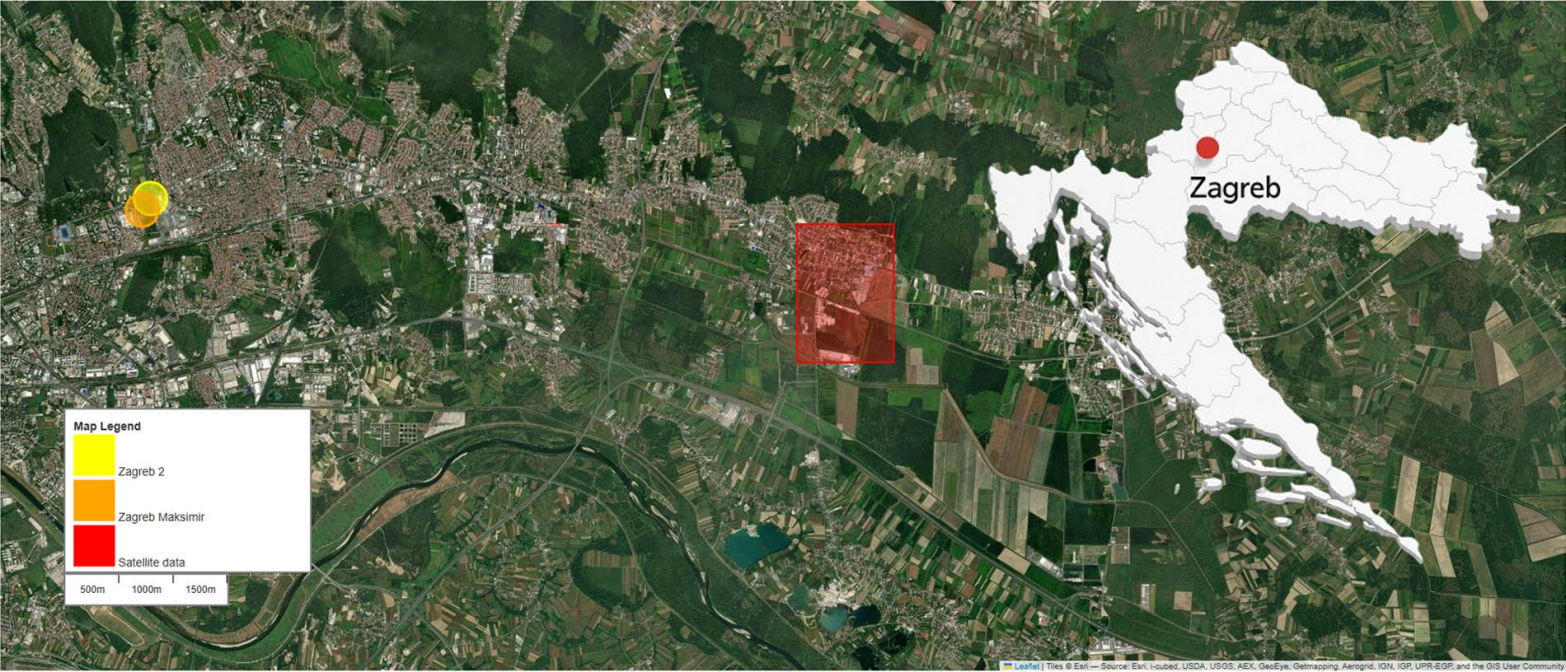
Locations of measuring sites and atmospheric data downloaded from satellites.

The flow rate is maintained at 1.0 ± 0.05 L/min with a fast response time of under 1 second for 90% concentration changes. In addition to data from outdoor low-cost sensors, two more outdoor data sources were used. First, data from two measuring sites from the Croatian Meteorological and Hydrological Service (CMHS) were incorporated. These two sites are the closest to the study location and are labeled with blue and green circles in Fig. 2. Additionally, data from the ERA-5 dataset (https://cds.climate.copernicus.eu/datasets/reanalysis-era5-single-levels?tab=overview), which provides hourly data from 1940 to the present, was obtained from the Climate Data Store (CDS) platform, explained in more detail in our previous work^28^. CDS provides the state of the atmosphere captured from satellites. For this study, an area is needed; we used the smallest area, with a house in the middle of it. The study’s location is within the red square shown in Fig. 2.

In addition to the hourly and minute-based data, the mass concentration of PM_1_ particle fraction was also collected on quartz filters by Air Metrics Mini Vol Sampler with a flow rate of 5 L min^-1^ (Airmetrics, Springfield, USA) for seven days. Two devices were used: daily measurements, running from 7 AM to 7 PM, and night measurements from 7 PM to 7 AM. PM_1_ concentration was determined gravimetrically. Concentrations of following PAHs: fluoranthene (Flu), pyrene (Pyr), benzo(a)anthracene (BaA), chrysene (Chry), benzo(j)fluoranthene (BjF), benzo(b)fluoranthene (BbF), benzo(k)fluoranthene (BkF), benzo(a)pyrene (BaP), dibenzo(ah)anthracene (DahA), benzo(ghi)perylene (BghiP), and indeno(1,2,3-cd)pyrene (IP) were determined by Agilent Infinity high-performance liquid chromatography (HPLC) with a fluorescence detector (Agilent Technologies, Santa Clara, CA, USA). Different measurements were also taken over shorter periods, such as airflow within the room. This was measured using the Testo 405i smart probe (manufacturer), which was moved around the room to capture airflow. A detailed description of this experiment can be found in the experiments section of this paper. Similarly, VOCs were sampled using Universal tubes (Markes International) by SKC pocket pump (SKC Limited, UK) with a flow rate of 30 mL min^-1,^ from 10 to 20 minutes. The VOC concentrations were performed using thermal desorption (TD) and gas chromatography with tandem mass spectrometry (GC-MS) (Agilent Technologies, Santa Clara, CA, USA). Tubes with samples were heated in a desorber up to 320 °C, and a helium flow rate of 50 mL min^-1^ was applied. A more detailed explanation of this analysis is also provided in the experiments section.

## Data Records

The data is stored in the EDIAQI project Zenodo repository at https://zenodo.org/records/14763137.

The final dataset is composed of 19 subsets. Six subsets are from LCS, one from the referent device, and two from auxiliary sensors for motion and door opening. Human motion inside the room and traffic are captured in separate subsets. The auxiliary data includes door data with 975 points and motion detection with 711 values, and these two datasets are combined. Outdoor data is stored in 4 subsets: two from low-cost sensors, one from meteorological sites in Zagreb, and one from satellite data. The smallest subset is from airflow measurements. In addition to the subsets, there are Black Carbon and CPC datasets. The Black Carbon dataset has 33,637 values, measured every minute with three columns, while the CPC dataset has 916,840 points measured every second with five columns. The airflow data contains only 498 values, recorded every second with two columns.

Door and motion sensor data are captured on every change, with 975 and 711 entries, respectively; one has three columns, and the other has 1. The indoor referent device has 35,441 values, recorded every minute with 20 columns. Each low-cost sensor has different lengths and columns, but all measure pollutants and ambient conditions. The first indoor provider has 28,387 values, recorded every minute with seven columns; provider 2 has 2,882 values, recorded every few minutes with nine columns. Sensor provider 3 has 28,773 values and six columns, while provider 4 has 524,560 values, recorded every 5 seconds, with 30 columns, where 15 columns represent one sensor. Provider 5 has four sensors, each with eight columns and 11,491 values recorded every 10 minutes. The last low-cost sensor dataset has 28,704 values every minute, with 10 columns for 12 devices. The outdoor data subset contains eight devices, 96 columns, and 964 rows, recorded every 30 minutes. The second outdoor sensor has 6,780 values, recorded every 5 minutes with 17 columns. The Zagreb measurement site data has 31 columns and 851 values captured every hour. People data is recorded every minute with 32,321 rows. Satellite data has 28 columns and 1,176 rows, measured every hour. Traffic data is captured with four columns and 6,593 rows, recorded every time a vehicle passes the house.

### Experiments

The study aims to gather data across a variety of real-life scenarios which were conducted:

- Candles burning
- Air diffusers in action
- Use of gas stoves
- Human presence and activity, including exercising on a rowing machine
- Car exhaust from the garden
- Cleaning activities
- Opening and closing of doors and windows

The events are documented with precise timestamps, while sensors and cameras collect data every minute, allowing a detailed analysis of how each event affects air quality.

The events are documented with precise timestamps, while sensors and cameras collect data every minute, enabling a detailed analysis of how each event affects air quality. This section thoroughly explains each experiment, with names corresponding to those in a JSON (JavaScript Object Notation) file attached to the data. The JSON file contains the exact start and end times for each experiment. Furthermore, Table 2 shows each experiment’s start and end times, the total duration, and the total number of minutes the experiments ran. Hence, all events recorded are given, and one can follow this in the provided data. Before starting each experiment, the room was empty, with no people present. If any residual effects of indoor pollution were detected beforehand, the room was ventilated by opening the doors to the garden to simulate ventilation and infiltration of external air.

**Table 2:** Shows all events, their start and end times in the Zagreb time zone, and the duration of each experiment.

Experiment	Start Date	End Date	Duration (Minutes)
Door opened to the Garden	16.10.2024 9:15	16.10.2024 9:47	32
Door opened to the Hallway	16.10.2024 20:55	16.10.2024 21:25	30
Doors opened to the Garden and the Hallway	17.10.2024 18:20	17.10.2024 18:50	30
Exercise Rowing with closed door to the Garden	19.10.2024 10:40	19.10.2024 10:54	14
Exercise Rowing with opened door to the Garden	19.10.2024 11:24	19.10.2024 11:41	17
Human Presence (1)	19.10.2024 12:02	19.10.2024 12:53	51
Human Presence (2)	19.10.2024 13:34	19.10.2024 14:08	34
Diffuser with Water inside	19.10.2024 14:33	19.10.2024 14:51	18
Diffuser with Oil inside	19.10.2024 15:06	19.10.2024 15:20	14
Car working in Garden, distance 3 m	19.10.2024 15:33	19.10.2024 15:38	5
Car working in Garden, distance 5 m	19.10.2024 15:53	19.10.2024 15:58	5
Gas Burner working with closed door	20.10.2024 13:10	20.10.2024 13:40	30
Gas Burner working with opened door	20.10.2024 14:00	20.10.2024 14:30	30
Candle with closed door (1)	20.10.2024 14:51	20.10.2024 15:15	24
Candle with closed door (2)	1.11.2024 14:50	1.11.2024 15:05	15
Scented Candle	20.10.2024 19:58	20.10.2024 21:30	92
Candle with opened door	21.10.2024 8:50	21.10.2024 9:30	40
Vacuum Cleaning with windows closed	26.10.2024 16:26	26.10.2024 16:34	8
Odor from Neighbors	29.10.2024 10:20	29.10.2024 10:25	5
Heater (1)	29.10.2024 21:00	29.10.2024 21:30	30
Heater (2)	31.10.2024 14:30	31.10.2024 15:00	30
Heater (3)	3.11.2024 10:40	3.11.2024 11:30	50
Aquarium Pump	31.10.2024 15:20	31.10.2024 15:40	20
Tar (Neighbor Burning, unplanned event during this time)	31.10.2024 15:56	31.10.2024 16:01	5
Central Heating	31.10.2024 17:45	31.10.2024 19:45	120
VOC Tubes - Car (1)	29.10.2024 11:02	29.10.2024 11:22	20
VOC Tubes - Car (2)	29.10.2024 11:46	29.10.2024 11:56	10
VOC Tubes - Diffuser (Oil) (1)	29.10.2024 12:28	29.10.2024 12:52	24
VOC Tubes - Diffuser (Oil) (2)	29.10.2024 12:52	29.10.2024 13:07	15
VOC Tubes - Gas Burner	29.10.2024 13:24	29.10.2024 13:39	15
VOC Tubes - Candle (1)	29.10.2024 14:20	29.10.2024 14:51	31
VOC Tubes - Candle (2)	29.10.2024 14:51	29.10.2024 15:06	15
Diffuser with Oil repeated	2.11.2024 16:30	2.11.2024 17:05	35
Boiling Water (Gas) with doors closed	2.11.2024 18:15	2.11.2024 19:45	90
Number of experiments	33		1004

For instance, during the gas burner experiment, the doors were opened to remove CO₂ and PM caused by the gas burner as a pollution source. After ventilation, the room was left undisturbed for 15 minutes to allow the sensors to stabilize at an equilibrium point, ensuring each experiment began under consistent baseline conditions. The experiments started with tests to observe how outdoor air and air from the rest of the house affect indoor air quality in the experiment room. The first experiment, titled **“Door opened to the Garden,”** involved opening the garden doors to monitor the effect of outdoor air on the indoor environment. In the next experiment, **“Door opened to the Hallway,”** the hallway doors were opened while the garden doors were closed to assess the influence of air from the rest of the house. Finally, the last test in this series, **“Doors opened to the Garden and the Hallway,”** created a draft by simultaneously opening both doors to evaluate the combined effects. For the remaining experiments, the doors to the garden were the primary variable being opened or closed, while the hallway doors remained open by default. In subsequent references to “doors,” this refers explicitly to the garden doors.

Two exercise experiments were conducted to assess the impact of human activity on indoor air quality. In one, a person exercised using a rowing machine with the doors closed; in the other, the same activity was performed with the doors open. Additionally, the effect of human presence was tested by having a person work inside the room on a computer. Following the human activity tests, experiments were conducted using diffusers. Two tests were performed: one with water inside the diffuser and another including essential oil. To investigate the impact of vehicle emissions on indoor air quality, a car was placed in the garden with the exhaust pointed towards the doors at distances of 3 and 10 meters. Next, experiments were run to evaluate how burning affects IAQ. These included tests with regular candles, a gas burner, and boiling water on the gas burner. Tests were conducted for all burning-related experiments with the open and closed garden doors. A simple camping gas burner was tested with and without a pot of water placed on top of it for boiling. Two types of experiments were performed to study the effects of heating: one with central heating via a radiator and another with a small electric convection heater placed inside the room.

Additionally, as the room contains an aquarium, a test was conducted to determine whether the aquarium pump, which aerates the water, contributes to indoor air pollution. During testing, neighbors burned materials twice, producing black smoke and a strong odor. These events were also timestamped for analysis. Some tests, such as those involving the car, diffuser, candles, and gas burner, were repeated to collect additional measurements using VOC tubes. The duration of the experiments varied, ranging from 5 minutes for the car emissions test to over an hour for tests involving human presence and heating.

#### VOC analysis

VOCs were collected using universal tubes containing a porous polymer, graphitized carbon black, and carbonized molecular sieves (Markes, Llantrisant, United Kingdom). VOCs were determined using a thermal desorption coupled with a gas chromatography/mass spectrometry (TD-GC/MS) system. Samples tubes were heated up to 320 °C in a thermal desorber in a helium flow. GC capillary column (6% cyanopropyl/phenyl, 94% polydimethylsiloxane, diameter 0.32 mm, film thickness 1.80 µm, length 60 m) was used for separation. The following 19 VOCs were identified: 2-methylpentane, methylene chloride, methylcyclopentane, chloroform, 2-methylhexane, cyclohexane, benzene, heptane, trichlorethylene, methylcyclohexane, toluene, tetrachlorethylene, ethylbenzene, m-p-o xylene, styrene, 1,3,5-trimethylbenzene, 1,4-dichlorobenzene.

## Usage Notes

**Fig. 3:**
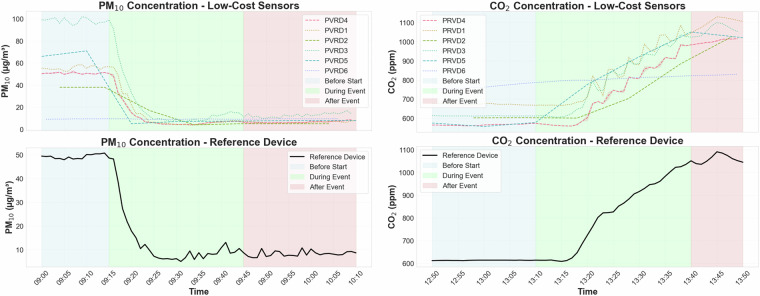
Concentrations of PM_10_ and CO₂ during two events: opening the door to the garden and gas burning.

To better understand the data, we provide some insights into the data collected in this study. Figure 3 shows how sensors reacted to two events. The first event was opening the door to the garden, during which PM_10_ measurements changed. For this showcase, every low-cost sensor is included in the figure with its values before, during, and after the event. In addition to PM_10_, CO_2_ levels were presented during a different event of gas burning. This figure illustrates how the sensors compare to the reference device and how responsive they are to each event. The sensors in this figure are the same for both events, meaning the hardware setup remains unchanged. The data wasn’t cleaned or preprocessed. There are two notebooks for downloading and loading the data in Python and R. The notebooks create a dictionary that loads all the data available in this study and does the initial preparation of the data.

## Data Availability

The data is available in the EDIAQI project Zenodo repository at https://zenodo.org/records/14763137. The code used to generate and load the data can be found at GitHub: https://github.com/valentinopetric/collocation_study.
